# Experiences and preferences of patients visiting a head and neck oncology outpatient clinic: a qualitative study

**DOI:** 10.1007/s00405-017-4453-7

**Published:** 2017-01-28

**Authors:** Jeroen A. S. Bisschop, Fabienne R. Kloosterman, Janneke E. van Leijen-Zeelenberg, Geert Willem Huismans, Bernd Kremer, Kenneth W. Kross

**Affiliations:** 1 Faculty of Health, Medicine and Life Sciences, Maastricht University, Maastricht, The Netherlands; 2 Department of Health Services Research, Maastricht University, Maastricht, The Netherlands; 3 Department of Otorhinolaryngology, Head and Neck Surgery, Maastricht University Medical Center, Maastricht, The Netherlands

**Keywords:** Patient preferences, Oncology, Qualitative research, Patient-centered care, Head and neck

## Abstract

The objective of this study is to report on an in-depth evaluation of patient experiences and preferences at a Head and Neck Oncology outpatient clinic. A qualitative research design was used to determine the experiences and preferences of Head and Neck Cancer patients in an Oncology Outpatient Clinic, Maastricht University Medical Center, The Netherlands. Head and Neck Cancer Patients, treated for at least 6 months at the Oncology Clinic, were included. A qualitative research design with patient interviews was used. All interviews were recorded and transcribed verbatim to increase validity. Analysis was done with use of the template approach and qualitative data analysis software. Three of the six dimensions predominated in the interview: (1) respect for patients’ values, preferences and expressed need, (2) information, communication and education and (3) involvement of family and friends. The dimensions physical comfort; emotional support; coordination and integration of care were considered to be of less significance. The findings from this study resulted in a deeper understanding of patients’ experiences and preferences and can be useful in the transition towards a more patient-centered approach of health care.

## Introduction

Within the medical community there is a tendency to create a higher quality of delivery of care. The American National Academy of Medicine (NAM), formerly known as the Institute of Medicine (IoM), is one of the leading nonprofit institutions in the world operating in the field of medicine, technology and science. The NAM provides objective and independent advice in this field. In advising policy makers, health professionals and the public at large with evidence-based authoritative information, the NAM aims to improve healthcare at large. To conceptualize a high quality of care, the NAM has stated several aims. These aims entail “safety, effectivity, patient centeredness, timeliness, efficiency and equity” [1].

One of the aims, the concept of patient-centered care (PCC), has gained increasing prominence in recent years as a key aim of healthcare delivery. As set by the NAM, the definition of PCC entails “Providing care that is respectful of and responsive to individual patient preferences, needs, and values and ensuring that patient values guide all clinical decisions” [1].

At the core of PCC lies the healing relationships between clinicians, patients and family members [2, 3]. In a patient-centered approach, interactions between clinicians and patients follow a two-way share of information [4, 5]. Clinicians help patients and their families make clinical decision and facilitate appropriate care. This is especially important in the context of cancer-related care. In oncology, the PCC approach often enables patients to follow through with invasive treatments or behavioral changes needed to improve health [6]. This approach aims to improve clinical practice by building relationships that bridge differences between doctors and patients [2, 3, 6]. In this way patients can be seen in context of their own social environment.

In practice, it is noted that PCC is an important factor in creating a care unit in which there is more patient involvement, which on its turn can increase the efficiency of care. Improvements in efficiency of care can be seen in less frequent patient visits, less scans, gathering of more information and, therefore, reaching a diagnosis sooner [7, 8].

At the core of patient centeredness is the idea that healthcare providers and the systems in which they work will deliver care that is attentive to the needs, values and preferences of patients [9]. For physicians, the most important tool for making care more patient centered is communication [10].

Having PCC as one of the main aims of care delivery, medical expertise is not the only role of the clinician anymore; achieving patient understanding of the situation and following shared decision-making too plays an important role. Furthermore, it is the additional care that clinicians have to fulfill such as referrals to palliative care and management of side effects that contribute to achieving a PCC model [1, 11].

The six dimensions described by the NAM are (1) respect for patients’ values, expressed preferences and needs, (2) information, communication and education, (3) coordination and integration of care, (4) emotional support, relieving fear and anxiety, (5) physical comfort and (6) involvement of friends and family [1]. In this study, qualitative in-depth analyses have been used to investigate these dimensions in detail. Qualitative in-depth analyses have shown to be a valuable addition to existing patient satisfaction questionnaires and other quantitative methods in the measurement of PCC. More specifically the use of qualitative analysis can lead to deeper insights and facilitate improvements.

Optimal quality and delivery of PCC in the treatment of Head and Neck Oncology (HNO) is very important. The HNO patient group differs from the average patient group seen at the otolaryngology (ORL) department in certain personal characteristics and complexity of care. The complexity of care needed for HNO patients relies on tumor location, cancer progression and impact on the activities of daily living. As seen in previous studies the most common side effects of HNO cancer treatment are the impact on physical appearance, speech and food ingestion [7, 9, 12]. Moreover, the need to adjust for risk factors that are known to cause head and neck cancer are complicating its care delivery. Risk factors include tobacco smoking, the consumption of alcohol and infections with Human Papilloma Virus [13]. The literature describes that head and neck cancer patients are mainly men, excessive smokers and alcohol abusers. Delayed medical care seeking is often the result of fear of doctors, having no pain and no suspicion of cancer [14–16].

Despite growing recognition of the importance of PCC, patients’ preferences and experiences within the group of HNO patients are less studied.

In this study, we aim to investigate the experiences and preferences within a group of HNO patients at the Oncology Center of Maastricht University Medical Center (MUMC). This study tries to fill the gap in existing knowledge surrounding PCC by systematically mapping patient preferences and experiences.

## Materials and methods

### Design

A qualitative research design was used to determine the experiences and preferences of Head and Neck oncology patients visiting the Oncology Center.

### Sampling

Patients were only included if they were treated at the MUMC Oncology Center for at least 6 months, in this way patients were experienced with the center and the type of care provided. Patients who received palliative treatment were excluded due to the different pathways of follow-ups and appointments regarding treatment. This could in some cases be difficult to compare with other respondents’ pathways.

Two researchers scanned the schedule and selected eligible patients. After giving consent for both the interview and the recording interviews were taken straight after consultation. Doctors were not informed about participation of the patients to keep them blinded.

### Data collection

Nineteen semi-structured interviews with patients were conducted. The interview questions were based on the six dimensions of PCC as referred to by the NAM [1]. The interview guide was pilot tested on intelligibility, interpretation and integrality by two volunteers before the start of the study. For each dimension, patients were asked to share their experiences and state their future preferences and expectations. Interviews were held by two trained researchers and let the participants elaborate on the topics. The two researchers were not involved in any part of the patient care and had not met the patients prior to the interview.

### Analysis

All interviews were recorded and transcribed verbatim to increase validity [17, 18]. The six dimensions of PCC were used as a model for coding. With use of the template approach the answers of the respondents were placed into a data matrix using NVivo software based on concepts of the codebook [19].

The complete ad verbatim answers (‘thick descriptions’) were transcribed by both researchers into boxes with only the most essential information (‘thin descriptions’) [20]. Next, the thin descriptions were transcribed into summaries per concept by two researchers independently. The summaries were discussed until consensus was reached. Finally, the answers of all respondents were compared with each other to search for possible discrepancies, similarities and deeper insights.

The Medical Ethical Commission of the MUMC+ concluded that full consideration of the study design was unnecessary under Dutch Law. To ensure quality of study reporting the COREQ checklist was used [21]. This checklist consists of 32 items divided into three domains that are described to evaluate the methodological robustness of qualitative studies (see Table 1).

## Results

In total, 49 patients were approached and 23 patients agreed to participate (response rate 47%). The 26 patients that did not agree to participate were not systematically questioned about their reasons and, therefore, reasons for non-response are not known. Three patients dropped out after approval, two received a bad diagnosis at the day of the interview and one did not show up. Patients’ characteristics are summarized in Table 2. The duration of the interviews varied between 18 and 61 min. An overview of the main results is presented in Table 3.

**Table 1:** Six dimensions of patient-centered care (PCC) [1]

Dimension	Definition
Respect for patients’ values, preferences and expressed needs	Healthcare is patient centered when recognizing and treating patients in an atmosphere in which they kept informed regarding their medical condition and get involved in decision-making
Information, communication and education	In PCC, information on diagnosis, prognosis and treatment is trustworthy and tailored to individuals
Coordination and integration of care	PCC ensures accurate information transfer and transitions to other settings
Emotional support and alleviation of fear and anxiety	PCC reacts to fear and anxiety associated with illness. These can be as debilitating as the physical effects. Caregivers should pay attention to both the patient and one’s family
Physical comfort	PCC provides tailored support to pain and other discomfort
Involvement of family and friends	In PCC, friends and family of the patients are supported as caregivers, respected and welcomed in the clinical setting

**Table 2:** Characteristics of respondents (*n* = 19)

Nr.	Sex	Age	Relevant history	Reason of visit^a^
1	M	63	cT1N0M0,pT1N0Mx SCC^b^ oral cavity, cT1N0M0, pT1N0Mx SCC oropharynx, cT1N0M0,pT1NxMx glottic larynx carcinoma	CWNR
2	V	62	cT4bN0M0, pT4bcN0Mx SCC ethmoid	Control a.t
3	M	67	rcT4N0M0, rpT4N0Mx cutaneous SCC meatus acusticus externa	Control a.t
4	M	81	cT1N0M0, pT1NxMx supraglottic larynx carcinoma	Control a.t
5	V	59	rcTxN3M0, pTxN3Mx in transit metastasis of melanoma of the head and neck area	Control a.t
6	M	63	cTisN0M0, pTisNxMx carcinoma in situ from the glottic area	Control a.t
7	M	76	cT2N0M0, pT2N0Mx SCC of the oral cavity	Control a.t
8	M	78	cT3N0M0, pT3N0Mx SCC of the parotid gland	Control d.t
9	M	70	cT1aN0M0, pT1NxMx glottic larynx carcinoma	Control a.t
10	M	59	cT2aN0m0, pT2N3Mx melanoma of the head and neck area	Control a.t
11	M	61	cT1bN0M0 glottic larynx carcinoma	CWNR
12	M	65	cT1aN0M0 glottic larynx carcinoma	Control a.t
13	V	67	cT0N2bM0, pT0N2bMx cutaneous SCC of the head and neck	Control d.t
14	V	58	cT2N1M0, pT2N0Mx SCC of the oral cavity	Control a.t
15	M	66	cT4N0M0, pT2NxMx cutaneous SCC of the vestibulum nasi	Control a.t
16	M	70	cT2N0M0, pT2NxMx acinic cell carcinoma of the parotid gland	Control a.t
17	V	71	cT1N0M0, pT1NxMx muco-epidermoid carcinoma of the parotid gland	Control a.t
18	V	80	cT4aN0M0 SCC of the maxillary sinus (operation refused by patient)	Control a.t
19	M	53	cT2N0M0, pT1NxMx chondrosarcoma of the subglottic area	Control a.t

^a^ *A.t*. after treatment, *D.T*. during treatment, *CWNR* consult with new results

^b^Squamous cell carcinoma

**Table 3:** Overview of results

Dimension	Results (number of patients)
Respect for patients’ values, preferences and expressed needs	Appointments are planned well (8) Patient had a say in the planning of the appointment (9) Patient did not have a say in planning of appointments (4) Combinatory appointments were planned, instigated by the administrative staff (13) Combinatory appointments were planned, instigated by patient (1) No combinatory appointments were planned (2)
Information, communication and education	Patient expressed own treatment preferences (8) Patient did not receive a choice in treatment (7) Communication was experienced as good/excellent (13) Good explanations by the specialists (8) Communication through leaflets (6) Communication through internet (3) People are helpful and respectful (1) Communication experienced as variable (3) General practitioner received information from HNO Oncology Center (8) General practitioner did not receive any information (11) Internal communication up to date (11) Internal communication was not sufficient (3) Personal questions were always answered (17)
Coordination and integration of care	Organization was considered good/excellent (11) Waiting times vary but were acceptable (11) Patient expressed a need for more privacy in the waiting room (2)
Emotional support and alleviation of fear and anxiety	Emotional support was offered but not accepted (12) Emotional support was offered and accepted (3) Emotional support was not offered (3)
Physical comfort	Facilities of the center considered as very good (1) No further improvement waiting room necessary (7) Waiting room needs further improvement regarding privacy (1) Physical contact with doctor was pleasant and comfortable (16)
Involvement of family and friends	Family was involved in the consults (19)

### Respect for patients’ values, expressed preferences and needs

Within this dimension, the majority of the respondents had a positive experience regarding the satisfaction about how their preferences were taken into account. Preferences for specific days and timeslots of appointment were specifically mentioned here.

In addition, with the exception of one respondent, all felt free to ask questions during consultations and felt included in decision-making about their care. Four respondents stated that personal priorities for treatment were even elicited and considered in multidisciplinary meetings. Five respondents reported explicitly that they had to show assertiveness to get their questions answered.

Within this dimension three respondents reported negative experiences stating that this was either due to the scheduling of their appointments, or was based on the feeling that the treatment was not being explained according to their level of knowledge.

Some respondents were given the opportunity to choose between the treatment methods (e.g. surgery versus radiation/chemotherapy) and felt free to express their personal preferences. The ones that did not receive this opportunity reflected this to either there not being another treatment option or wanting the doctor to decide ‘as he knows best’.

## Information, communication and education

Within the dimension Information, Communication and Education, five main topics emerged. In general, respondents were satisfied with the doctor–patient communication.

Communication is important in explaining complicated information to this group of patients. One of the respondents emphatically mentioned the impact of receiving an unfavorable diagnosis and the rush of the steps that had to be made:

The moment you are diagnosed with cancer I do not think one can think clearly. I can imagine people being totally lost in such situations. The doctor said: ‘we can operate you’. That is a really big thing. I think they should sometimes be more restrained in the way they express themselves. (Respondent no. 16).

First, the communication with the administrative staff was described as respectful with a problem-solving approach with the patient being a central part.

Second, internal communication between the HNO department and other medical specialists was generally experienced as being up to date.

All doctors were informed about my story, my operation and the whole process. Three surgeons operated on me. There were many different specialists and for me it was one team. I cannot complain. (Respondent no. 1).

Internal miscommunication within the HNO department emerged as third topic and was mentioned by three respondents, mainly in situations in which a locum physician was in charge who was not up to date with the patient’s dossier.

Well I once had a doctor that replaced my own physician, but he didn’t know anything and I never have to see him again; but that is the only minor miscommunication I have had. (Respondent no. 7).

Fourth, with exception of two respondents, all reported that they received sufficient information about their situation in the form of leaflets, websites or verbal explanation. With the exception of two respondents, sufficient education was provided about the kinds of toxicities and expected adverse effects. There were two patients who specifically desired more information on prognosis and rehabilitation but they acknowledged the uncertainties of the prognosis and future of their disease. One respondent preferred to be given information in the format of reliable internet websites with treatment-specific information which was not provided by the specialist.

External communication was the final topic to emerge from the interviews and here mainly negative feedback was provided by the respondents. More specifically, the communication with the general practitioner generally occurred months after treatment. Some of the communication was given to the wrong person or was non-existent.

### Coordination and integration of care

Within the dimension coordination and integration of care, four main topics emerged. With regard to planning, respondents experienced flexibility in the planning system, both for single appointments and for combinatory appointments with different specialisms planned on one day.

In general, respondents provided positive experiences with care coordination and waiting times on the day of the appointment were considered to be acceptable.

When answering the questions regarding this dimension most respondents answered similarly, mentioning their understanding of the severe situations all patients go through when visiting the Oncology Center. Therefore, patients can relate and reflect to each other and have the upmost respect for prolonged waiting times. As these patients are diagnosed with a certain type of cancer, this reflects in the understanding of prolonged waiting times because they understand why a consultation might be taking longer, either to explain new results or to get a better understanding of the diagnosed cancer.

Respondents were asked their opinion on waiting room information screens showing waiting times and delays. All but one respondent did not consider this idea to be of added value and some even mentioned it to be in conflict with their privacy.

One respondent said:

I would not prefer a screen like that at this Oncology Center; I do not think it is appropriate. There are always impatient people, but if you realize that this time is miniscule compared to a lifetime, then what is the problem? As for me no extra facilities like screens have to be made. (Respondent no. 6).

All respondents reacted positively regarding access times for treatments; they felt they did not have to wait long.

The most prominent negative experiences noted were due to seeing different doctors at subsequent appointments.

We had an appointment with our doctor, but then we received a message that a new doctor was scheduled to help us that day. We really did not like that, especially because he had to tell us new test results and the prognosis. There was no explanation; they only told us our regular doctor was absent. (Respondent no. 12).

### Emotional support-relieving fear and anxiety

Thirteen respondents reported that emotional/psychological support was not necessary; all stated that the possibilities were explained by the doctors. Four respondents and one partner were given support by a social worker. The respondents and partners who received help from a social worker experienced this as a positive experience.

I have had four appointments with the social worker, and if I needed more it could be arranged. I could count on her, in the beginning I did not think I would need it, but at some point I thought well why not, it is being offered and I could use the help. (Respondent no. 5).

### Physical comfort

Two results can be derived within the dimension of physical comfort. Some respondents reported that their physical comfort in the waiting room is of minor importance in comparison to the reason why they were treated.

Other respondents mentioned this from another perspective that due to fact that they are all diagnosed with cancer, more space is needed for privacy.

Look, all people in that room have cancer. From simple, to really severe and complex cases. I can feel that. And because you sit there with six in a row, it would be helpful to have a bit more space. (Respondent no. 6).

This privacy is considered as important. As another respondent puts it:

The waiting room has to be more separated. I think there are people who do not like to be seen there at all. Myself, I sometimes have that feeling, especially when you are very sick. (Respondent no. 16).

The respondents all noted to have had positive experiences with the physical comfort during physical examinations performed by the doctor.

Nine respondents explicitly mention the nervousness with which they enter the consultation room. Eight of them were calmed down by caring attitudes of the specialists. Exploration of the patient’s management of the symptom burden showed mixed results. Three respondents explained that they were given practical tips and tricks to ease symptoms but most considered the disease burden (pain, dysphagia, xerostomia, fatigue, etc.) as part of the diagnosis and accepted them without further questioning.

### Involvement of family and friends

With respect to the involvement of family and friends, almost all respondents reported positive experiences. In general, respondents felt that family and friends receive equal attention during the appointment. If that was not the case, two respondents reported that their partner was partly ignored during consultations; they would make their preferences clear. As a partner of one of the respondents put it:

We always visited the center together. As a couple we can pick up more details the doctor tells us. And if the things I say are not taken into account, I become assertive and make clear my wife and I are in this together. Who says I cannot be with her? (Respondent no. 2).

The involvement and respect to the opinions and worries of friends and family was considered very important for the majority of patients.

### Summary of results

First, three dimensions of PCC predominated the interviews: (1) respect for patients’ values, expressed preferences and needs, (2) information, communication and education, (3) involvement of family and friends. Within these dimensions, patients attached specific importance to three aspects: provision of honest and complete information; an open discussion on the decision-making with involvement of the patient and considering affection with family and friends as a crucial part in the treatment.

The dimensions physical comfort, emotional support-relieving fear and anxiety and coordination and integration of care were of less significance according to the patients. However, comforting nervous patients was considered as crucial for a specialist in this field. Within the coordination of care remarkably low attention was given to waiting times on the day of appointment.

## Discussion

Despite the growing attention for PCC and the recognition of its importance to HNO patients, relatively little is known about the specific experiences and preferences of this group of patients. Based on the results derived from this study, specific insights are provided.

These findings differ from those of a recent study that explored patients’ experiences and preferences with PCC at a general outpatient otorhinolaryngology (ORL) department [22]. In that study waiting times played a more essential role in the overall patient experience. This implicates that compared to the ORL department, the coordination of patient care is organized differently at the Oncology Center. In general, the coordination and planning covers more complex cases that need several appointments and patients expect the waiting times to be longer, while the coordination and personal planning at the ORL department cover less complexity and personal planning.

Second, assertiveness was seen as an important factor in this study and in the literature. Patients’ lack of assertiveness in cancer care can challenge implementing PCC into practice [23, 24].

Third, within the HNO outpatient clinic attention is given to the emotional support, either on a professional note or within their own personal circle. This is in contrast to the outpatient ORL department, where none or only little attention is perceived to be given [22]. A majority of the respondents in this study did not need the professional overall support; however, attention to the emotional side of the illness was positively received.

Last, the involvement of family and friends is of great significance. The significance of involving family and friends is in line with the findings at the outpatient ORL department and in cancer care worldwide [22, 25, 26].

The main findings of this study can be systematically implemented in clinical practice. (1) The information transfer can be optimized during care transitions. Multiple other studies support the value of coordinating information transfer between hospital, home, and other care delivery settings [27–29]. (2) Consultant and patient should agree on a mutually acceptable communication strategy. When asked, patients made known their preferred communication strategies: some prefer email, others periodic in-person visits or telephone calls. Neglecting patient preferences for communication risks additional care fragmentation. (3) Social support seems to be important in the care of oncology patients. Clinicians can link patients with community resources and services. These services can decrease isolation and may improve engagement and patient cooperation [30].

### Limitations

Using qualitative research methods has some limitations for generalizability and validity of the research findings. Because the reasons for non-response were not systematically asked, there is a chance of positive bias. It should be noted that findings from this study might be influenced by the context and place of the interviews. The findings might, therefore, not be directly transferable to other settings. Part of this limitation is found in the concept of PCC, as being subject to the specific needs of a patient. In addition, a recent study has shown that it is possible to use these types of specific patient experiences on an aggregate level to optimize care delivery [31].

Finally, some interesting differences between patients are not examined in this study and could be influential to personal perception of healthcare; for instance, the expectations on emotional support for patients in which the cancer altered their (facial) appearance versus patients without visible alterations.

## Conclusion

This study provides an insight into patients’ experiences and preferences at a Head and Neck Oncology outpatient clinic. Certain dimensions of PCC within the HNO patient population differ from those in the patient population at the discussed ORL outpatient at the same hospital.

This indicates that it is important to determine which preferences are specific for a certain group of patients. One has to make clear which characteristics determine such preferences. An interesting subject for further research would be to question if a certain patient profile can be made and if health care can be more specified and improved based on such a profile. This is an important discussion point in the transition towards a more patient-centered approach of health care.
